# Adapting and Developing an Academic and Community Practice Collaborative Care Model for Metastatic Breast Cancer Care (Project ADAPT): Protocol for an Implementation Science–Based Study

**DOI:** 10.2196/35736

**Published:** 2022-07-25

**Authors:** Ashley J Housten, Uzoma Charles Okere, Graham A Colditz, Cynthia Ma, Jingxia Liu, Courtney Harriss, Nancy U Lin, Melissa Rooney, Jennifer Dill, Muhammad Popalzai, Jennifer Badiu, Kan Huang, Casey Burton, Lindsay Peterson

**Affiliations:** 1 Division of Public Health Sciences Department of Surgery Washington University School of Medicine St. Louis, MO United States; 2 Department of Medicine Washington University School of Medicine St. Louis, MO United States; 3 Department of Medicine Harvard Medical School Boston, MA United States; 4 Missouri Baptist Medical Center St. Louis, MO United States; 5 SIH Cancer Institute Southern Illinois Healthcare Carterville, IL United States; 6 Phelps Health Delbert Day Cancer Institute Rolla, MO United States

**Keywords:** metastatic breast cancer, care coordination, Project ADAPT, referral process, implementation science, oncology, community practice, academic institutions, breast cancer, cancer, breast, implementation, science

## Abstract

**Background:**

Metastatic breast cancer (MBC) remains incurable despite significant treatment advances. Coordinating care for patients with MBC can be challenging given the various treatment options, available clinical trials, and frequent need for ancillary services. To optimize MBC care, we designed a project for adapting and developing an academic and community practice collaborative care model for MBC care (Project ADAPT), based on the Ending Metastatic Breast Cancer for Everyone (EMBRACE) program developed at Dana Farber Cancer Institute.

**Objective:**

We aim to describe the implementation science–based study design and innovative components of Project ADAPT.

**Methods:**

Project ADAPT uses the Dynamic Adaptation Process informed by the Exploration, Preparation, Implementation, Sustainment framework. Washington University School of Medicine (WUSM) partnered with 3 community hospitals in the St. Louis region covering rural and urban settings. The exploration and preparation phases provide patient and provider feedback on current referral practices to finalize the approach for the implementation phase. At the implementation phase, we will enroll patients with MBC at these 3 community sites to evaluate potential collaborative care at WUSM and assess the impact of this collaborative care model on referral satisfaction and acceptability for patients with MBC and their providers. Patients may then return to their community site for care or continue to receive part of their care at WUSM. We are incorporating virtual and digital health strategies to improve MBC care coordination in order to minimize patient burden.

**Results:**

The exploration phase is ongoing. As of August 2021, we have recruited 21 patient and provider participants to complete surveys of the current collaborative care process at WUSM. Using a 2-tailed paired *t* test, 44 patients (including 10 patients from the exploration phase) and 32 oncologists are required to detect an effect size of 0.5 with 80% power at a level of significance of .05. Throughout this phase and in preparation for the implementation phase, we have iteratively updated and refined our surveys for the implementation phase based on testing of our data collection instruments. Our partner sites are in various stages of the single institutional review board (IRB) approval process. We have ongoing engagement with all partner sites, which has helped solidify our participant recruitment strategies and design patient-friendly recruitment materials. In addition, we have included a patient advocate on the research team. Members of the research team have launched a single IRB Support Network at WUSM to create a repository of the single IRB procedures in order to streamline the partner site onboarding process and facilitate enhanced collaboration across institutions.

**Conclusions:**

With this robust model, we expect that patients with MBC will receive optimal care regardless of geographical location and the model will improve patient and provider experiences when navigating the health system.

**International Registered Report Identifier (IRRID):**

DERR1-10.2196/35736

## Introduction

### Background

As of 2019, there were over 3.8 million breast cancer survivors in the United States, with this number projected to increase substantially by 2030 [1]. Excluding skin cancers, breast cancer is the most common cancer type and accounts for 30% of new cancer diagnoses in the United States [2,3]. In 2021, breast cancer incidence was expected to be 284,200 in the United States, with a 0.5% annual increase [3]. Although mortality rates have declined, the projected number of deaths from breast cancer in 2021 was over 44,000, and nearly all these deaths were due to metastatic breast cancer (MBC), which has a median survival of about 3 years [3,4]. Breast cancer incidence is higher in urban populations than among those living in rural settings [5]. However, this rural-urban disparity has been attributed to lower mammography screening rates, due to limited access to health care services in rural settings and low socioeconomic status [6,7], impacting this population’s ability to obtain quality care and making the diagnosis of breast cancer at later stages more likely [8]. When viewed at the population level, these barriers often explain the observed gap in breast cancer incidence between urban and rural populations [7].

Despite significant advances in treatment, there is no cure for MBC [9-12]. Current treatment options are palliative in nature with the goal of extending survival and improving quality of life, but are frequently encumbered by care coordination challenges [9,11,13]. Even with national guidelines for the treatment of MBC and subtype-specific MBC, which encourage participation in clinical trials [14-16], there is not always a clear sequence of treatments or an accessible clinical trial available for patients, particularly those in rural settings [9,17-19]. Additionally, there is often a need for ancillary supportive care services for patients with MBC [17,19]. The complexities involved in the routine care of patients with MBC can lead to underutilization or overutilization of care; missed opportunities to improve cancer outcomes, including cancer-specific survival and treatment-related symptoms; and undue patient and care delivery burden. Therefore, there is a need for coordinated care models that cater to the increasing prevalence of patients with MBC in the United States [20].

### Limitations of Current Care Models

Patients with MBC usually choose to receive cancer care near their homes. Treatment may be at a community or academic center. However, while many patients live near academic centers, a considerable number of patients also travel to academic centers for treatment or second opinions, clinical trial options, and ancillary services not available at their local community practice [21]. Effective coordination and communication between the referring provider and oncologists at academic centers are needed to maximize the benefits of these consultations. When coordination is lacking, patients may be seen at an academic center when they are not candidates for a clinical trial or when a relevant trial is unavailable. Treatment plans may also be delayed if certain medical records or up-to-date test results are unavailable. In addition, patients are often not referred due to referring physicians’ lack of access to real-time clinical trial options at an academic cancer center or their concern for patient burden, including financial responsibility, health insurance limitations, and transportation cost [17,19,22]. Given these referral barriers, communication is often limited to a phone call or email between individual physicians, which is not always a secure, reliable, or organized approach. This way of communication also inhibits collaborative consultations for patients who may benefit from a multidisciplinary review of their case. Therefore, existing communication media and channels lack efficiency and create barriers to eliciting a second opinion or screening patients potentially eligible for clinical trials. Ultimately, a lack of consistent uniform coordination and communication among physicians is likely to result in decreased patient satisfaction and potentially missed opportunities to improve patient outcomes [13].

Addressing physician-level barriers to referring patients, such as physicians’ lack of awareness of available trials, concern of losing patients, and lack of time [23], is imperative to adequate patient care. Additionally, current referral practices encompassing preappointment communication regarding available trials, required testing and records, patient functional status, and preferences must be honed among academic and referring community centers. A collaborative care model can improve workflow, minimize patient burden, improve care delivery and communication between physicians, and ultimately enhance the referral process between academic and community cancer centers [22].

In this protocol paper, we describe the implementation science study design of a project for adapting and developing an academic and community practice collaborative care model for MBC care (*Project ADAPT*). This project evaluates clinical health service utilization (eg, access and utilization of clinical trials, virtual consult/telemedicine, and genomic testing), and the fidelity and adoption of a coordinated care intervention between academic and community settings. This model can then be tested on a larger scale, with a more significant number of community partners, to evaluate its impact on additional outcomes, including quality of life, and progression-free and overall survival, among patients with MBC.

## Methods

### Study Development

#### EMBRACE Program

*Project ADAPT* originated from a review of the Ending Metastatic Breast Cancer for Everyone (EMBRACE) clinical program at the Dana Farber Cancer Institute (DFCI) [24]. The DFCI research team explicitly designed the EMBRACE program for MBC to (1) enhance the longitudinal care of patients with MBC; (2) develop a robust, seamless, collaborative care model between the DFCI and referring providers; and (3) improve the quality of life and satisfaction with care among patients with MBC. To accomplish these goals, EMBRACE created a clinical flow model that identifies patients with MBC seen at the DFCI (either newly diagnosed or recently seen at the DFCI), streamlines and coordinates the initial consultation, facilitates treatment at the DFCI or collaborative care between institutions, and coordinates reconsultations at the DFCI on disease progression. This process enables ongoing contact with the patient and between providers, and leverages the expertise of providers and health care administrators to achieve this care coordination for MBC [24].

Most medical oncologists at the DFCI who completed the initial baseline survey reported moderate satisfaction with the initial consultation and stated that access to the referring provider’s email was extremely important. They expressed dissatisfaction with the current approach of accessing the emails of referring providers. In addition, among referring providers, most reported difficulty in referring patients to academic institutions for clinical trials. Patients who completed the baseline survey at the DFCI strongly desired all aspects of information on their cancer and treatment [24].

With similar challenges in our local institution and community, and with this intervention in mind, we adapted existing knowledge and tools from EMBRACE into the design and implementation of a care model for the patients, providers, and local community cancer sites in and around St. Louis, Missouri by engaging several community practices and experts in implementation science and medical oncology. We named this intervention *Project ADAPT*.

#### Project ADAPT

Disparities in breast cancer stage at diagnosis have been observed in the St. Louis region among medically underserved patients and have been attributed to barriers in the referral pathway to Siteman Cancer Center (SCC), the only National Cancer Institute (NCI)-designated comprehensive cancer center within a 240-mile radius of St. Louis [25]. SCC comprises physicians and researchers from Washington University School of Medicine (WUSM), Barnes-Jewish Hospital, and St. Louis Children’s Hospital. Thus, SCC provides several vital resources for cancer research in the St. Louis metropolitan area and extends throughout the catchment area. Approximately 10%-15% of patients referred via private health care practices presented at late stages (stages III and IV) compared with about 40% of patients referred via Safety Net clinics, a referral system used by community clinics that cater primarily to uninsured and underinsured locals [25]. A collaborative care model has the potential to address these disparities. Existing collaborative care challenges identified by the research team include the need for (1) efficient and reliable communication between the referring community and accepting academic providers; (2) optimal timing of genomic testing to aid in decision-making for next-line therapies and access to clinical trials; and (3) minimizing patient burden (eg, excess travel and unnecessary in-person appointments).

The goal of *Project ADAPT* is to implement a multilevel collaborative model between academic and referring community oncology practices to accelerate the translation of evidence into practice to improve MBC management and the patient referral process. This multilevel model for coordinated care leverages the clinical expertise of oncologists at both the academic and partner institutions managing patients with MBC, the numerous clinical trials available at SCC, and the unique NCI Community Oncology Research Program (NCORP) resources at the community partner centers. Employing the single institutional review board (IRB) structure through the WUSM, *Project ADAPT* extends existing SCC partnerships in the St. Louis region to develop a sustainable collaborative care model [26]. To achieve this goal, we have the following specific objectives: (1) assess patients’ satisfaction and acceptability of the academic and community collaborative care model for their MBC care; (2) evaluate providers’ (academic oncologists and referring oncology providers) satisfaction and acceptability of the collaborative care model with the referral and management processes of MBC patients; and (3) evaluate the implementation of the adapted EMBRACE program using fidelity and adoption measures.

**Figure 1:**
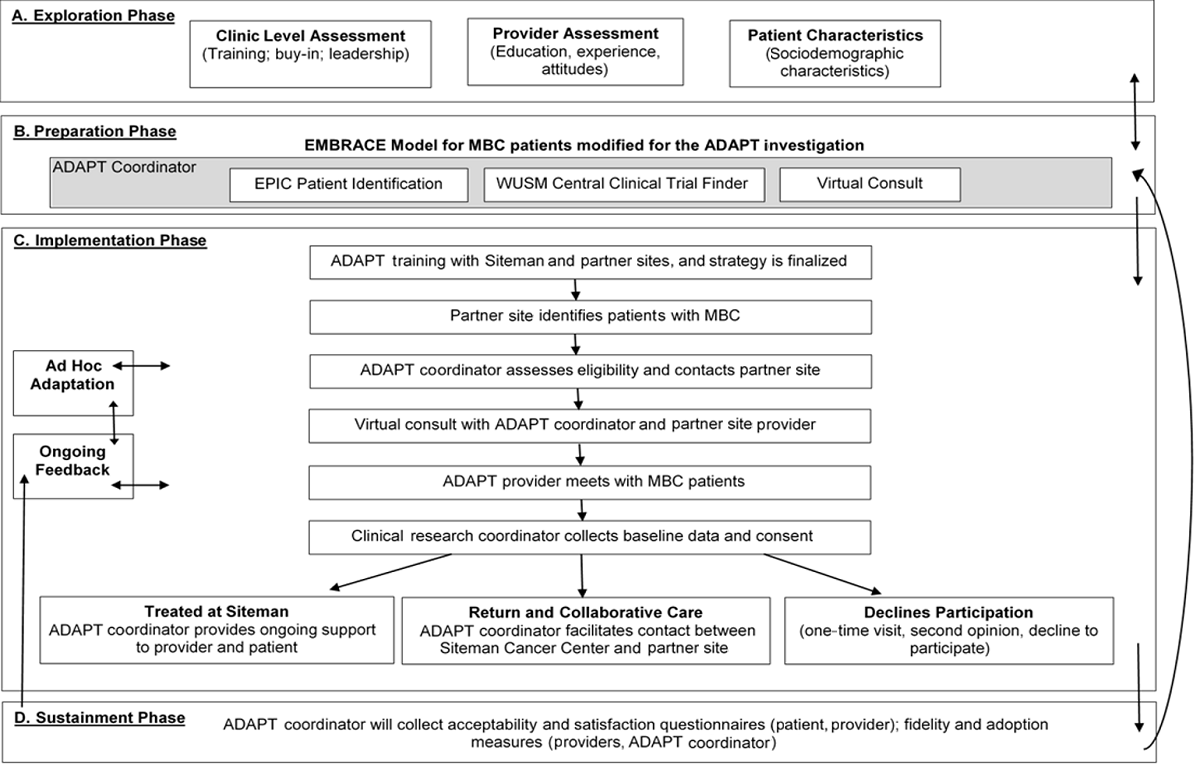
Research strategy. Figure adapted from the Dynamic Adaptation Process and Exploration, Preparation, Implementation, Sustainment framework. EMBRACE: Ending Metastatic Breast Cancer for Everyone; Project ADAPT: project for adapting and developing an academic and community practice collaborative care model for metastatic breast cancer care; MBC: metastatic breast cancer; WUSM: Washington University School of Medicine.

To accelerate the translation of evidence-informed MBC management across multiple systems into practice, we are using the dynamic adaptation process (DAP) to adapt EMBRACE to reflect the characteristics and context of the St. Louis regional care environment [27,28]. This approach builds upon the EMBRACE evidence-informed practice and modifies the intervention to fit the proposed collaborative environment of St. Louis. This adaptation includes the 4-phase Exploration, Preparation, Implementation, Sustainment (EPIS) model for multilevel program design [27,28]. This innovative implementation science framework allows us to assess our multilevel strategy by incorporating ongoing feedback to make modifications during the investigation, thereby enhancing our ability to intervene by identifying and testing real-time developments as needed to advance the pace of translating evidence into practice. Figure 1 shows the theoretical pathway of *Project ADAPT* using the DAP and EPIS implementation science framework [27,28].

### Ethics Approval

WUSM/SCC received single IRB approval in May 2021 from the Washington University Institutional Review Board (Protocol #202104173-1001). Due to the various characteristics of our 3 partner sites, these sites are in different stages in the administrative and registration processes to obtain single IRB approval through the WUSM IRB. Utilizing the single IRB approval process strengthens the existing executive partnerships to create a sustainable research collaboration [26].

### Study Settings

Washington University in St. Louis School of Medicine (WUSM) is partnering with the following 3 community hospitals within the St. Louis region to establish this care model: Missouri Baptist Medical Center (MBMC) in St. Louis County, Missouri; Southern Illinois Healthcare (SIH) in Carbondale, Illinois; and Phelps Health Delbert Day Cancer Institute (DDCI) in Rolla, Missouri. These hospitals are affiliated with broader regional health care organizations that offer the potential to expand and scale-up *Project ADAPT*. An existing informal referral relationship exists between each of these partner sites and SCC. Therefore, we are building off this foundational relationship to coordinate care for patients with MBC. Specifically, MBMC and SIH are affiliated with The BJC Collaborative, LLC, an organization inclusive of 7 health systems in Missouri and Illinois working together to enhance the quality of care, increase access to health services through meaningful population health benefits, and reduce the total cost of care within the Midwest region. Phelps Health is a county hospital serving South Central Missouri, whose Delbert Day Cancer Institute is affiliated with the Siteman Cancer Network, an affiliation led by SCC committed to improving the health and well-being of people and communities through research, treatment, and prevention. WUSM is the project’s lead/coordinating site.

The lead site, WUSM, has an extensive history of research, education, and patient care, as one of the preeminent medical research institutions in the United States. SCC is the only NCI-designated cancer center in Missouri and is part of the main Washington University Medical Center campus with multiple satellite locations throughout the St. Louis region. SCC brings together over 450 physicians and researchers from WUSM, Barnes-Jewish Hospital, and St. Louis Children’s Hospital (both part of BJC Healthcare). Thus, SCC provides several vital resources for cancer research in the St. Louis area, with extension throughout the SCC catchment area.

MBMC’s Cancer Center is a regional leader in providing care and support to patients throughout their cancer care. This cancer center aims to deliver treatment, comprehensive care, and advanced research to patients receiving care in the surrounding areas. The hospital has over 1700 new cancer cases each year, and the cancer center is staffed with 8 medical oncologists. MBMC has outreach sites in more rural areas in Missouri and Illinois, for example, MoBap Sullivan in Sullivan, Missouri, and is an active partner in this effort to share best practices and recruit women with MBC. In 2019, prior to the COVID-19 pandemic (as this has caused significant disruptions in cancer care), MBMC’s Cancer Center referred 3 out of 41 patients with MBC to SCC.

SIH is a leading health care system serving the people of southern Illinois. SIH offers services in rural clinics in addition to a regional referral center for the 16-county region. SIH aims to provide expertise and advanced treatments tailored for the needs of the rural setting. As the region’s first dedicated cancer treatment center, the SIH Cancer Institute provides surgical and treatment options using evidence-based approaches to advance the care of patients with MBC. There are 5 medical oncologists, 2 radiation oncologists, and 1 breast surgical oncologist in the cancer program serving an estimated 200 breast cancer patients yearly. From 2016 to early 2019, out of the 26 patients with MBC seen at SIH, there were no referrals to SCC.

The DDCI provides cancer care services and continuity of care for patients in a rural 7-county region across South Central Missouri. The DDCI offers radiotherapy, medical oncology, laboratory, clinical research, and imaging services. All cancer treatment and support services are at a single location to ensure the best possible patient experience and to ease the burden of traveling to multiple locations for treatment. In addition, it offers ancillary services, such as patient education and customized counseling services, to achieve the mission to improve the health and wellness of people in the region. The Phelps Health DDCI had 10 patients with MBC, with only 1 referral made to SCC, in 2019.

### Study Population

In the exploration phase, participants must meet the following inclusion criteria: (1) diagnosis of MBC (stage IV); (2) referral to SCC for MBC treatment; and (3) referral before the implementation phase of *Project ADAPT*. For the implementation phase, the inclusion criteria are (1) histologically or cytologically confirmed MBC (this includes newly diagnosed [de novo] or recurrent metastatic disease); (2) age ≥18 years; (3) self-reported ability to speak and understand English; and (4) willingness to provide written informed consent. There are no participant limitations based on sex.

For this protocol paper, providers recruited for this study are oncologists who primarily manage MBC cases and are involved in the referral process of patients with MBC. We have used the term “provider” throughout the protocol to make it inclusive for other sites that may replicate this study and have clinical buy-in from a variety of health care providers. The inclusion criteria for oncologists are as follows: (1) academic oncologists from SCC and oncologists from participating partner community sites, and (2) willingness to provide written informed consent. Table 1 shows the distribution of participants throughout the study phases.

**Table 1:** Participants involved in the study phases.

Phase	Patients	Providers
Exploration	A subset of patients with metastatic breast cancer referred to Siteman Cancer Center (SCC) before the implementation phase (n=10)	Oncologists from 3 community hospitals and oncologists at SCC (n=32)
Preparation	None recruited (patient advocate included as part of the research team)	Same group as in the exploration phase (including research team)
Implementation	Newly diagnosed (de novo) patients or patients with recurrent disease referred to SCC after the exploration phase (n=34)	Same group as in the exploration phase
Sustainment	Patients recruited in the implementation phase	Same group as in the exploration phase

### Study Components

*Project ADAPT* has 5 intervention components designed to enhance communication between providers, continue engagement between research and clinical teams, and provide optimal patient care regardless of geographic location. These components are as follows: (1) ADAPT coordinator; (2) Epic communication between providers and research teams; (3) Powerful partnerships with collaborating sites; (4) WUSM clinical trial finder; and (5) Virtual consult/telemedicine.

#### ADAPT Coordinator

The ADAPT coordinator is the facilitator and intermediary between the NCI-designated SCC and our 3 community hospitals within the NCORP. The ADAPT coordinator handles all operations for the study to run smoothly. The ADAPT coordinator disseminates information to all sites and handles recruitment procedures, survey distribution, and data collection. The ADAPT coordinator also leads the strategy and standardizes the coordination for Epic patient identification and utilization of the clinical trial finder, as well as virtual consults between providers/telemedicine for patients. Specific training, including On Core training, Epic training, and research training, and a background in medicine, public health, or a health-related field to review clinical trial eligibility information may be beneficial for this role.

#### Epic Communication Between Providers and Research Teams

Hospitals in the United States are required to use an electronic medical record (EMR) system to manage patient health information [29,30]. Epic is a type of EMR system that enables customization and versatility across various hospital settings. The secure chat feature within Epic is the potential communication tool intended for study team communication. The use of streamlined Epic communication processes between community and academic center physicians and research staff is designed to improve patient and provider satisfaction, enable efficient consultation, and enhance clinical outcomes. In this adaptation, we aim to leverage existing technology to facilitate communication between the referring sites and SCC. Thus, we identified an electronic communication option, such as Epic, since oncologists were currently using this software to manage patients. When considering reproducing this method for sites using different EMR software or when scaling up to other sites with differing EMR software in the future, leveraging existing electronic communication channels, like secure group email or Microsoft Teams, may be worth exploring through acceptability and feasibility measures prior to selecting a communication channel.

This study relies on a uniform strategy for providers to obtain consults on patients with MBC via a previsit virtual consult that is tracked and readily available in the EMR (ie, Epic), significantly improving patient care and coordinating communication between providers. Thus, we have identified community sites that use Epic to facilitate communication. This process simplifies referrals and enhances information security, as this communication does not occur over email or phone calls, but instead through Epic.

#### Powerful Partnerships With Collaborating Sites

Partnerships with our 3 community hospitals offer access to a care network with an electronic health record (Epic) and diverse provider systems to study wide-ranging management of patients with MBC. This care model is designed as a bidirectional partnership since the community sites have access to NCORP resources. We aim to connect patients from SCC to these resources to enhance admission and enrollment in clinical trials at both community sites and SCC. Also noteworthy is that these 3 partner sites serve rural patient populations with MBC and are located across the St. Louis region, representing the breadth of care settings within the area.

#### WUSM Clinical Trial Finder

The WUSM clinical trial finder is a website that displays available clinical trials at WUSM and SCC. The WUSM clinical trial finder allows the ADAPT coordinator to identify clinical trials for which patients at partner sites may be eligible. The ADAPT coordinator facilitates coordination and communication with the treating oncologists and clinical team members to develop a detailed treatment plan for the patient. In addition, the ADAPT coordinator conducts virtual demonstrations and training to teach community sites the best approaches to navigate the website and clinical trial tree.

#### Virtual Consult/Telemedicine

The virtual health care feature of *Project ADAPT* consists of virtual consults between providers and telemedicine for patients. Virtual consults between providers at partner sites are designed to discuss potential referrals and clinical cases through secure Epic communication. These virtual meetings allow for discussion of patient matters and determination of clinical trial availability and eligibility for patients between oncologists at SCC and community partner sites. This digital strategy will enhance care delivery and reduce patient burden by preventing potentially unneeded in-person screening as traditionally carried out.

The second virtual feature is telemedicine. While there are multiple definitions of telemedicine, telemedicine generally applies digital media/platforms to aid the clinical decision of providers in the management of patients [31,32]. This strategy is designed for patients to receive care remotely through various virtual communication tools [33]. The emergence of COVID-19 has caused a large increase in telemedicine use throughout the US health sector in cancer care and primary health services [34,35]. *Project ADAPT’s* telemedicine component affords a patient access to specialist care from SCC without the need for an in-person visit. This approach covers patients who need care but cannot travel to SCC. It can also reduce patient time and financial burden if consultation can be provided virtually rather than as traditional in-person visits, preventing unnecessary in-person consultations.

### Study Measures

Several surveys employed within this study explore patients’ and providers’ satisfaction with the referral process and are grouped into patient and provider surveys. Surveys designed for the patient population are as follows: (1) ADAPT patient survey; (2) ADAPT patient sociodemographic survey; (3) Epic data extraction form; and (4) Decision Regret Scale [36] (Multimedia Appendices 1-5).

The ADAPT patient survey (Multimedia Appendix 1) captures patients’ satisfaction and acceptability of the current referral process. There are open-ended and free-response options that offer participants the opportunity to provide insights and experiences valuable to the study. The ADAPT patient sociodemographic survey (Multimedia Appendices 2 and 3) includes baseline characteristics of patients with MBC referred from outside institutions to SCC to gain insights into the backgrounds of our patient sample population. The Epic data extraction form (Multimedia Appendix 4) gathers information on tumor characteristics, treatment history, and receptor status from Epic. The Decision Regret Scale [36] (Multimedia Appendix 5) evaluates patients’ feelings of regret (if any) regarding transferring cancer care from partner sites to SCC.

Surveys for the providers recruited into *Project ADAPT* are as follows: (1) ADAPT provider survey and (2) Implementation Climate Scale [37] (Multimedia Appendices 6-8). The ADAPT provider survey (Multimedia Appendices 6 and 7) emphasizes providers’ satisfaction and acceptability of the current referral process, and the Implementation Climate Scale [37] (Multimedia Appendix 8) captures the level of application of evidence-based health practices at the partner sites. These surveys also include free-response options that delve deeper into providers’ encounters with the referral system.

### Study Phases/Procedures

#### Exploration Phase: Gathering Information and Recruitment

In the exploration phase, the ADAPT coordinator identifies approximately 10 patients through Epic who have been previously referred to SCC from external institutions (to the breast oncologists at SCC involved in this study). These patients are contacted via phone to inquire if they are willing to participate in this phase of the study. If they agree to participate, they consent during the phone call and complete the ADAPT patient survey and sociodemographic questions through the Research Electronic Data Capture (REDCap) link [38,39] sent to their email addresses or over the telephone.

Oncologists at SCC and the partner sites are involved during this study phase. The research team discusses the proposed ADAPT process through virtual meetings and refines the recruitment process from feedback during these meetings. These virtual meetings facilitate continued buy-in and leadership from referring providers to champion the intervention at their sites. In addition, providers at partner sites and SCC are recruited by email, provide consent, and then can complete the ADAPT provider survey through REDCap links sent to their email addresses.

The following privacy protections are enacted for all email communications involving protected health information: (1) emails are sent securely (ie, [secure] in the subject line); (2) the body of the email instructs the participant to send all information as a response to this thread and to not remove “[secure]” from the subject line; and (3) the participant’s agreement to provide information over email is documented in our research records. Multimedia Appendix 9 outlines the recruitment processes of the exploration phase in a flow diagram.

#### Preparation Phase: Procedures

Research subjects are not recruited during this phase. Data collected during the exploration phase guide the preparation phase strategies, including how the ADAPT coordinator leads the process for facilitating the following: (1) patient identification for the implementation phase; (2) WUSM central clinical trial finder; and (3) virtual consult/telemedicine. There is ongoing engagement with oncologists and the research team at both SCC and community sites to incorporate modifications as needed at this study phase, as illustrated in Figure 1.

#### Implementation Phase: Recruitment and Procedures

The recruitment process can be done in 2 ways. The partner site clinical/research team members identify patients with MBC, and they contact the ADAPT coordinator who screens the patients with MBC for eligibility or the ADAPT coordinator identifies an eligible patient at a partner site through granted access to the Epic database through the following processes. The partner site provides a list of participating providers’ clinic schedules and hospital names on Epic. According to the oncologists’ schedules, the ADAPT coordinator maps out weekdays to screen providers’ lists of scheduled patients before their appointment day in Epic. The ADAPT coordinator then contacts the partner site provider (or the provider’s clinical team) scheduled to see the patient, alerting the provider of the patient’s eligibility status and available ADAPT resources (eg, ADAPT coordinator, clinical trial finder, and virtual consultation/telemedicine). The clinical/research team members at the partner site discuss the options with the patient. If interested, the patient can provide consent to either the research team at the partner site or a SCC site research coordinator (including the ADAPT coordinator). The patient is treated at SCC or declines (eg, declines participation or decides to be treated at the referring facility). Patients can refuse to participate and continue treatment at their preferred location.

These recruitment strategies are designed to accommodate our partner sites’ different human and material resources to provide a seamless recruitment process. Partner sites can either have the ADAPT coordinator lead the recruitment process of screening and identifying eligible participants through the detailed process outlined above while the research team obtains consent from the patients at the clinic through REDCap, or decide to oversee the recruitment process as the best fit for their respective centers and obtain consent from the patients at the clinic through REDCap. There must be regular communication between the ADAPT coordinator and the delegated recruitment research staff at these centers to evaluate or modify these processes if needed.

For those patients who provide consent, the ADAPT coordinator collects acceptability and satisfaction surveys at enrollment and at 3 and 6 months. During the implementation phase, providers complete the ADAPT provider survey and the Implementation Climate Scale [37] at baseline and at 3 and 6 months through REDCap. The providers recruited from the 3 community hospitals and at SCC in the exploration phase are the same providers involved at this stage, so they would not need to provide consent again. Multimedia Appendix 10 outlines the flow of the recruitment process.

#### Sustainment Phase: Recruitment and Procedures

The acceptability and satisfaction surveys collected at enrollment and at 3 and 6 months provide the feedback needed to adapt the implementation strategy (Figure 1). Our conceptual framework is a dynamic implementation approach to incorporate ongoing feedback to inform ad hoc adaptation. Therefore, we can modify the implementation strategy during our data collection process. During the sustainment phase, the ADAPT coordinator will complete adoption and fidelity observation measures to ensure the sustainment of the intervention. In addition, modified satisfaction and acceptability measures will be administered to patients and providers to continue evaluating and adjusting the program as needed.

### Statistical Analysis

#### Quantitative Analysis

Only a subset of patients previously referred to SCC (n=10) who complete surveys in the exploration phase will require descriptive statistics for the measures completed.

The Epic data extraction form measures are collected at T0 and T2 in the implementation phase. Specifically, clinical or pathologic stage and the date of diagnosis of metastatic disease are collected at T0 only. Receptor status is collected at T0 for all the enrolled patients and at T2 for patients with a subsequent biopsy. The number of prior and current lines of therapy are collected at T0 and T2 for all the enrolled patients. The measures at T0 and T2 are shown in Table 2.

**Table 2:** Patient assessment tools/schedule.

Measures	Exploration (0-3 months)^a^	Preparation	Implementation phase^b^		
			T0	T1 (3 months)	T2 (6 months)
Epic data extraction form	Yes	No	Yes	No	Yes
Sociodemographic survey	Yes	No	Yes	Yes^c^	Yes^c^
ADAPT^d^ patient survey	Yes	No	Yes	Yes	Yes
Decision Regret Scale [36]	No	No	No	Yes	Yes

^a^Only a subset of patients previously referred to SCC (n=10) is consented to complete surveys before the implementation phase begins.

^b^Patients enrolled in the implementation phase represent new patients referred from our partner sites to SCC for treatment.

^c^We are capturing changes to already provided sociodemographic information from enrolled participants, such as changes in insurance provider status.

^d^ADAPT: adapting and developing an academic and community practice collaborative care model for metastatic breast cancer care.

For other measures collected at multiple time points (eg, sociodemographic questions), a generalized estimating equation (GEE) model with appropriate link function is used to analyze the longitudinal data. The correlation among the repeated measures from the same participant needs to be considered. We are using an autoregressive of the first order as a working correlation structure, and patients with missing values at any time points are excluded from the GEE analysis. The GEE model includes time points. The GEE model’s *P* values from type 3 analysis are used to assess whether the outcomes across all time points are different. The least-square means for each outcome at each time point are then estimated. The standard errors are calculated using the GEE sandwich method when accounting for within-patient correlation. All analyses are conducted using SAS (SAS Institute) at the 2-sided 5% significance level. To prioritize improving satisfaction and acceptability across educational groups, racial/ethnic groups, or diagnosis stage, a subgroup analysis by race/ethnicity to identify any potential similarities or differences in responses is conducted.

#### Qualitative Analysis

A minimum of 2 research team members will use conventional content analysis to analyze responses from the open-ended and free-response questions [40]. This approach is partially rooted in naturalistic inquiry to explicate patient experiences and perspectives [41]. Any discrepancies in the analysis will be resolved through consensus coding. If the 2 coders cannot reach a consensus, a third research team member will act as a tiebreaker. Topics derived from the content analysis are used to adapt and modify the implementation strategy. This mixed-methods approach allows for a more in-depth and rich description of patient acceptability and satisfaction to provide context to the quantitative data.

#### Sample Size Calculation

As this is a feasibility investigation, there is no pilot data. One purpose of this study is to obtain measures of central tendency and variability to inform power calculations for our future randomized controlled trial. Sample size estimates are dependent on the effect size, defined as the difference of T2 (6 months) and T0 (enrollment) divided by the standard deviation. Using a 2-tailed paired *t* test, 44 patients (including 10 patients from the exploration phase) and 32 oncologists are required to detect an effect size of 0.5 with 80% power at a level of significance of .05.

## Results

The lead site WUSM received single IRB approval in June 2021, and data collection commenced immediately. As of August 2021, the research team has completed participant recruitment for the exploration phase with 10 patients and 11 providers from SCC, who completed surveys for the exploration phase, as shown in Tables 2 and 3. The participating sites are still at various stages of the single IRB approval process, which involves a signed reliance agreement between institutions, site registration, and a *Project ADAPT* application process. Once approvals are granted, oncologists at these partner sites are to complete surveys for the exploration and preparation phases to be finalized, as outlined in Table 1. We have incorporated a patient advocate into the study to understand patient cancer care pathways for reaching and engaging with more patients at the community level.

**Table 3:** Provider assessment tools/schedule.

Measure	Providers involved	Exploration	Preparation^a^	T0^b^	T1 (3 months)	T2 (6 months)
ADAPT^c^ provider survey	Siteman and community/partner site providers	Yes	No	Yes	Yes	Yes
Implementation Climate Scale [37]	Siteman and community/partner site providers	No	No	Yes	Yes	Yes

^a^No survey is administered during the preparation phase.

^b^The implementation phase starts at T0, marking the enrollment of patient participants to the study, and ends at T2.

^c^ADAPT: adapting and developing an academic and community practice collaborative care model for metastatic breast cancer care.

## Discussion

### Overview

Our adapted coordinated care model can enhance relationships among academic and community cancer centers. This model has the potential to improve patient and provider satisfaction and acceptability of the cancer care referral process. We anticipate that patients and providers can help identify limitations with the current MBC referral process to help identify opportunities to improve satisfaction and acceptability. Through *Project ADAPT*, we aim to positively impact clinical trial enrollment for patients with MBC, improve case discussion among providers, and provide a feasible and sustainable solution for improving care among patients with MBC. Moreover, as the number of women living with MBC continues to grow, our research extends beyond existing advancements in treatment [20,42] to evaluate interventions that have the potential to improve quality of life. We plan to disseminate our findings through academic platforms (eg, conferences and peer-reviewed journal articles), and we plan to make our data publicly available while adhering to IRB protocols. In addition, we will disseminate our findings to our community partner sites in a consumer-friendly plain language format.

There are few studies on the impact of collaborations among different hospitals to improve MBC care coordination. Only the EMBRACE program, to our knowledge, has conducted a multilevel systemic care coordination model for patients with metastatic breast cancer. WUSM received IRB approval in June 2021 and has completed participant recruitment for the exploration phase. WUSM has recruited 21 participants, including patients and providers. The latest community hospital, DDCI, joined the project in March 2021, while MBMC and SIH onboarded in late 2020. All partner sites are going through the single IRB registration in accordance with the current single IRB policy of the National Institutes of Health for multisite nonexempt human research carried out in the United States. The aim is to enhance and standardize the IRB review process among multiple research centers, avoiding duplication of review efforts to allow research to begin promptly [43]. This single IRB infrastructure has the potential to enhance the sustainability of these research partnerships and collaborations.

All communication requires reaching out to key stakeholders at these partner hospitals, presenting our research idea, and requesting collaboration virtually via Zoom meetings. Continued partnership and engagement have remained this way with a digital media platform by scheduling regular monthly meetings with all team members via Zoom to discuss updates on the study, recruitment and planning strategies, and IRB approval status. The research team meets at a time when most, if not all, can attend, and the ADAPT coordinator later sends out meeting minutes to all members of the group via email. During the research team meetings, study process modifications include creating study materials, such as the ADAPT patient flier for distribution to eligible patients at clinics and a page summary of *Project ADAPT* for providers.

We are approved to conduct virtual consultation for providers to share resources and knowledge among SCC, an NCI-designated cancer center, and our partner sites with NCORP clinical trial resources, creating a bidirectional relationship that improves patient care. This virtual consultation runs concurrently with telemedicine for patients. This platform of providing care to patients with MBC has become even more critical with the current COVID-19 pandemic [35]. This virtual experience also captures patients who would not be able to travel to SCC for whatever reason, but could still receive quality specialist care through this medium.

Patient recruitment for the exploration phase is via telephone calls, and surveys are distributed electronically through REDCap links. Utilizing e-consent for *Project ADAPT* has made recruitment manageable, especially during the COVID-19 pandemic. It resonates with studies showing that this approach can enhance access to participation among rural cancer patients in clinical trials, which is usually difficult due to geographic location [19,44]. Moreover, through virtual demonstrations via Zoom, the ADAPT coordinator has shown partner sites how to navigate the clinical trial tree finder.

Use of the digital EMR Epic messaging tool for communication among providers streamlines the referral process and allows providers access to real-time information regarding available clinical trials at SCC. Finally, as we prepare for the implementation phase, we strive to improve this design model with input from experts in different fields participating in the study and lessons learned from each stage of this study.

### Limitations

We have buy-in and support from the leadership, oncology teams, and staff at the community partner sites. A minimum commitment of monthly scheduled communication throughout the duration of the project is expected, and continued engagement in the adaptation process will be needed from all sites. It is possible that the initial support and enthusiasm for this project will diminish, but we are using an implementation-science framework to enhance continued engagement. In addition, we are working with sites that use the Epic EMR system. The feasibility data collected in this investigation will give us a sense of the timeline, personnel, and community setting infrastructure, including the EMR system, needed for scaling up this type of study.

### Opportunities for Future Research

WUSM research team members have launched a single IRB Support Network to develop a shared repository of resources and information to help guide partner sites through the single IRB process. We are in the process of creating a toolkit of resources to illustrate, in plain language, the single IRB application process. This toolkit will create a shared database with information about existing single IRB relationships with external partners (eg, key characteristics about the site and the research infrastructure, existing IRB agreements with WUSM, and past/current studies collaborated on with WUSM) and help to determine outreach strategies that best support partner sites throughout the single IRB process. For example, the study team has found that smaller community sites often lack third-party accreditations for their clinical research programs that may be more commonplace for larger academic health centers. In this way, the single IRB application process for studies that include a wide variety of recruitment sites would benefit from a streamlined approach to ensure the protection of human research subjects while accommodating the unique capabilities of each site.

In preparation for a larger trial, our team will complete provider-focused Pragmatic Explanatory Continuum Indicator Summary-2 (PRECIS-2) [45]. Evaluating our approach using the 9 PRECIS-2 domains (ie, eligibility, recruitment, setting, implementation resources, provider strategy flexibility, intervention flexibility, data collection, primary outcome, and primary analysis) will facilitate engagement from our care coordination stakeholders to match our research approach with the overall study aims of a future trial. The implementation science methods used throughout *Project ADAPT* will establish a robust methodological foundation for future trials investigating care coordination across multiple sites.

## Appendix

Multimedia Appendix 1 Adapting and developing an academic and community practice collaborative care model for metastatic breast cancer care (ADAPT) patient survey.

Multimedia Appendix 2 Adapting and developing an academic and community practice collaborative care model for metastatic breast cancer care (ADAPT) patient sociodemographic survey (exploration and T0).

Multimedia Appendix 3 Adapting and developing an academic and community practice collaborative care model for metastatic breast cancer care (ADAPT) patient sociodemographic survey (revised for patients at T1 and T2).

Multimedia Appendix 4 Epic data extraction form.

Multimedia Appendix 5 Patient Decision Regret Scale.

Multimedia Appendix 6 Adapting and developing an academic and community practice collaborative care model for metastatic breast cancer care (ADAPT) Siteman provider survey.

Multimedia Appendix 7 Adapting and developing an academic and community practice collaborative care model for metastatic breast cancer care (ADAPT) referring provider survey.

Multimedia Appendix 8 Provider Implementation Climate Scale.

Multimedia Appendix 9 Recruitment procedures at the exploration phase.

Multimedia Appendix 10 Recruitment procedures at the implementation phase.

## Abbreviations

ADAPT: adapting and developing an academic and community practice collaborative care model for metastatic breast cancer care
DAP: dynamic adaptation process
DDCI: Phelps Health Delbert Day Cancer Institute
DFCI: Dana Farber Cancer Institute
EMBRACE: Ending Metastatic Breast Cancer for Everyone
EMR: electronic medical record
EPIS: Exploration, Preparation, Implementation, Sustainment
GEE: generalized estimating equation
IRB: institutional review board
MBC: metastatic breast cancer
MBMC: Missouri Baptist Medical Center
NCI: National Cancer Institute
NCORP: National Cancer Institute Community Oncology Research Program
PRECIS-2: Pragmatic Explanatory Continuum Indicator Summary-2
REDCap: Research Electronic Data Capture
SCC: Siteman Cancer Center
SIH: Southern Illinois Healthcare
WUSM: Washington University School of Medicine
