# Supplementary material for: Adapting and Developing an Academic and Community Practice Collaborative Care Model for Metastatic Breast Cancer Care (Project ADAPT): Protocol for an Implementation Science–Based Study
Source: JMIR Res Protoc. 2022 Jul 25;11(7):e35736. doi: 10.2196/35736 (PMC9361152; doi:10.2196/35736)
Supplement: Multimedia Appendix 1 [file resprot_v11i7e35736_app1.doc]

*Page 1*

This survey focuses on your experience with the referral process from your community hospital to Siteman Cancer Center (SCC) and your cancer care at both care centers.

Please complete the ADAPT patient survey below.

Thank you!

Date

|  | __________________________________ |
| --- | --- |
|  | (mm-dd-yyyy) |
|  |  |
| 1. What is the name of your community hospital? | Southern Illinois Healthcare |
|  | Missouri Baptist Medical Center |
|  | Phelps Health Delbert Day Cancer Institute |
|  | Other |
|  |  |
| 2. How is this survey being administered? | Online (Remotely) |
|  | In person (Hospital) |
|  |  |
| RA initials | __________________________________ |
|  |


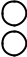

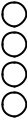


**Referral is the transfer of care of a patient from one clinician or clinic to another by request.**

| 3. | What is the name of the oncologist who referred you | __________________________________ |
| --- | --- | --- |
| to Siteman Cancer Center (SCC)? | |
|  |  |  |
| 4. | Who initiated the referral process? | I did |
|  |  | My doctor |
|  |  | Someone else |


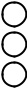


Why did you request to be referred?

|  | __________________________________ |
| --- | --- |
|  |  |
| Please who is this person to you? | __________________________________ |
|  |
|  |  |
| 5. Why were you referred from your community provider | Clinical trial |
| to Siteman Cancer Center (SCC)? | Discussion of best next line therapy |
|  | Management of side effects |
|  | Patient preference |
|  | Second opinion |
|  | Prefer to self-describe/Other |
|  |  |
| Please describe the reason for your referral to SCC | __________________________________ |
|  |
|  |  |
| 6. How did you find out about ADAPT study? | __________________________________ |
|  |


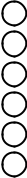


*Page 2*

**Thank you for agreeing to complete this survey today. Next, we are going to start with some questions about your cancer care.**

**On a scale from "not at all satisfied" to "extremely satisfied", kindly rate the following below**

| Not at all | Slightly satisfied | Moderately | Very satisfied | Extremely |
| --- | --- | --- | --- | --- |
| satisfied |  | satisfied |  | satisfied |

7. What is your overall


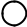

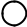

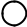

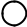

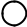


satisfaction with your initial

breast cancer care visit with

your doctor at your community

hospital?

8. What is your overall


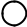

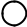

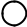

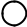

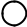


satisfaction with your follow up

visit with your doctor at Siteman

Cancer Center (SCC) for your

breast cancer care?

Why weren't you satisfied with the initial visit with

your doctor at your community hospital?

__________________________________________

Why weren't you satisfied with your visit at SCC?

__________________________________________

**On a scale from "not at all satisfied" to "extremely satisfied", how satisfied were you with the following below?**

| Not at all | Slightly satisfied | Moderately | Very satisfied | Extremely |
| --- | --- | --- | --- | --- |
| satisfied |  | satisfied |  | satisfied |

9. Your knowledge about


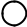

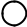

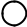

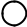

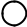


supportive care like counselling

services after your follow up visit

at SCC?

10. Your knowledge about


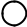

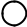

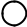

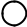

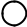


educational resources such as

brochures on drugs, diet and

exercise after your follow up visit

at SCC?

11. Your knowledge about


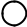

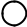

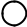

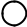

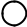


clinical trials after follow up visit

at SCC?

For any knowledge question, why were you not

satisfied?

__________________________________________

*Page 3*

**On a scale from "not at all important" to "extremely important", how important are the following services at Siteman Cancer Center (SCC)?**

| Not at all | Slightly | Moderately | Very important | Extremely |
| --- | --- | --- | --- | --- |
| important | important | important |  | important |

12. Cancer testing, ordering, and


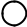

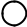

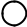

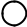

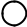


results

13. Clinical trials


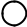

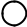

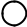

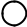

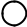


14. Supportive care such as


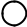

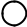

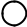

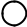

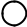


counselling services

15. Educational resources such


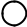

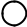

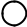

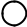

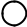


as brochures on drugs, diet, and

exercise

16. What other resources were important to you at SCC?

__________________________________________

**On a scale from "disagree" to "agree", how would you rate the following below about your referral experience. (Note: SCC is Siteman Cancer center)**

| Disagree | Neither agree nor | Agree | Don't know or Not |
| --- | --- | --- | --- |
|  | disagree |  | applicable |

17. I welcomed the idea of a


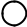

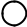

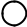

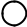


referral from my doctor at my

community hospital to SCC.

18. My doctor at my community


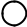

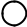

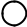

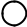


hospital seemed to support my

referral to SCC.

19. I felt informed about the


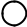

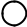

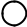

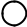


referral process from my

community hospital to SCC.

20. I felt the appointment at SCC


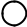

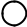

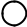

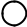


was scheduled in a reasonable

timeframe.

21. I felt the referral process


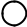

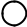

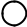

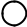


from my community hospital to

SCC was easy.

22. I felt the appointment with


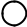

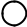

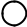

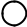


SCC worked with my schedule.

23. I trust the decision made by


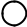

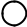

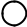

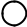


my doctor at my community

hospital in referring me to SCC.

*Page 4*

24. I was able to do necessary


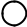

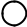

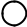

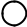


tests (like blood tests, scans or

biopsy) before I visited SCC, as

opposed to having to complete

them after I saw the SCC doctor.

25. I was adequately reminded


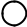

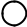

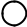

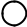


about my appointment before

my clinic day at SCC.

26. I felt my doctor at SCC was


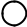

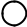

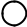

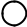


happy to receive my referral.

27. My doctor at SCC had the


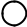

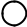

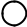

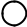


necessary test results (like blood

tests, scans or biopsy results)

needed to provide care before

my visit.

28. I felt my doctor at SCC took


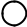

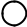

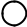

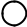


enough time to listen to me.

29. I felt my doctor at SCC took


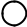

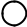

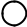


enough time to share some of

the available resources with me.

30. I felt I could contact my

doctor at SCC outside of an

appointment in case I had

questions.

31. I was comfortable with my

doctor at SCC.

32. I trust my doctor at SCC with

my cancer care.

33. My visit at SCC provided me

with helpful information about

my cancer care.

34. I was able to get travel

and/or lodging assistance to get

to SCC.

35. I had family or friends come

with me to my appointment at

SCC.

36. I had to pay additional out of

pocket costs after being referred

to SCC.

37. My health insurance paid the

additional costs (if any) from

being referred to SCC.

*Page 5*

38. After my visit at SCC, it was

clear what my next steps were

(for example, return to my

doctor at my community

hospital, start my care at SCC).

39. Overall, being referred to

SCC was beneficial to my care.

40. Overall, I liked the steps

taken in referring me from my

community hospital to my visit

at SCC.

1. Based on your experience, how can this referral process be improved?

__________________________________________

You did not select an option or provide an answer to a question(s) above, do you wish to continue?

Yes

No
