# Supplementary material for: Adapting and Developing an Academic and Community Practice Collaborative Care Model for Metastatic Breast Cancer Care (Project ADAPT): Protocol for an Implementation Science–Based Study
Source: JMIR Res Protoc. 2022 Jul 25;11(7):e35736. doi: 10.2196/35736 (PMC9361152; doi:10.2196/35736)
Supplement: Multimedia Appendix 2 [file resprot_v11i7e35736_app2.doc]

*Page 1*

This is the ADAPT study. There are a few surveys to be completed. The next survey will automatically load when to click "submit" at the bottom of each survey.

We want to know your experience with the referral process and cancer care from your community hospital to Siteman Cancer Center (SCC) and how best you think the overall process can be improved to guide future referrals.

In this survey, we are asking questions about you so we can get a better understanding of the people completing this survey.

Thank you!

Date of survey

|  | __________________________________ |
| --- | --- |
|  | (mm-dd-yyyy) |
|  |  |
| 1. Date of birth | __________________________________ |
|  |
|  | (mm-dd-yyyy) |
|  |  |
| 2. Gender | Female |
|  | Male |
|  | Non-binary |
|  | Prefer to self-describe |
|  | Prefer not to answer |
|  |  |
| Prefer to self-describe my gender as | __________________________________ |
|  |
|  |  |
| 3. Race (you can select more than one option) | Anglo American/Euro American/White |
|  | African American/Black |
|  | Asian American |
|  | Native Hawaiian or other Pacific Islander |
|  | Native American or Alaska Native |
|  | Prefer to self-describe |
|  | Prefer not to answer |
|  |  |
| Prefer to self-describe my race as | __________________________________ |
|  |
|  |  |
| 4. Hispanic or Latinx origin | Yes |
|  | No |
|  | Prefer not to answer |
|  |  |
| Ethnic background (you can select more than one | Mexican/Mexican American |
| option) | Central American |
|  | South American |
|  | Cuban |
|  | Puerto Rican |
|  | Spanish |
|  | Prefer to self-describe |
|  | Prefer not to answer |
|  |  |
| Prefer to self-describe my ethnicity as | __________________________________ |
|  |


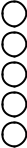

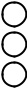


*Page 2*

| 5. Health Insurance | Medicaid |
| --- | --- |
|  | Medicare |
|  | Employer-based insurance |
|  | Tri-Care |
|  | Union-based |
|  | Self-pay (purchase insurance on own) |
|  | Uninsured (no insurance) |
|  | Prefer to self-describe |
|  | Prefer not to answer |
|  |  |
| Prefer to self-describe my health insurance as | __________________________________ |
|  |
|  |  |
| 6. Relationship status | Single |
|  | Married |
|  | Divorced |
|  | Widowed |
|  | Living with significant other |
|  | separated |
|  | Prefer not to answer |
|  |  |
| 7. Do you have a primary care physician? | Yes |
|  | No |
|  | Prefer not to answer |
|  |  |
| 8. Primary language spoken at home | English |
|  | Spanish |
|  | Prefer to self-describe |
|  | Prefer not to answer |
|  |  |
| Please specify the primary language you speak | __________________________________ |
| at home |
|  |  |
| 9. What level of formal education have you completed? | 8th grade or less |
|  | Some high school (HS) |
|  | Graduated HS or GED |
|  | Vocational school |
|  | Associate degree |
|  | Bachelor's degree |
|  | Graduate or professional degree |
|  | Prefer not to answer |
|  |  |
| 10. What is your employment status? | Full time |
|  | Part time |
|  | Retired |
|  | Unemployed |
|  | Prefer not to answer |
|  |  |
| 11. Do you have more than one job? | Yes |
|  | No |
|  | Prefer not to answer |
|  |  |
| How many jobs do you have? | __________________________________ |
|  |


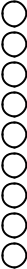

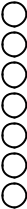

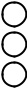

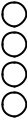

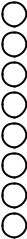

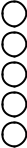

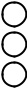


*Page 3*

| 12. What is your yearly family income, including all | Less than $15,000 |
| --- | --- |
| sources? | $15,000 -$34,999 |
|  | $35,000-54,999 |
|  | $55,000-$74,999 |
|  | $75,000 or more |
|  | Prefer not to answer |
|  |  |
| 13. Would you say that in general your health is | Excellent |
|  | Very good |
|  | Good |
|  | Fair |
|  | Poor |
|  | Prefer not to answer |
|  |  |
| 14. How often do you have someone help you read | All the time |
| hospital materials? | Most of the time |
|  | Some of the time |
|  | A little of the time |
|  | None of the time |
|  | Prefer not to answer |
|  |  |
| 15. How confident are you filling out medical forms by | Extremely |
| yourself? | Quite a bit |
|  | Somewhat |
|  | A little bit |
|  | Not at all |
|  | Prefer not to answer |
|  |  |
| 16. How often do you have problems learning about your | All the time |
| medical condition because of difficulty understanding | Most of the time |
| written information? | Some of the time |
|  | A little of the time |
|  | None of the time |
|  | Prefer not to answer |
|  |  |
| 17. What is your email address? | __________________________________ |
|  |
|  |  |
| You did not select an option or provide an answer to a | Yes |
| question(s) above, do you wish to continue? | No |


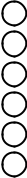

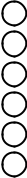

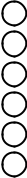

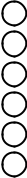

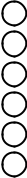

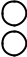


References

Questions 14-16 are the Brief Health Literacy Screener

Chew LD, Bradley KA, Boyko EJ. Brief questions to identify patients with inadequate health literacy. Fam Med. 2004 Sep;36(8):588-94. PMID: 15343421.
