# Supplementary material for: Adapting and Developing an Academic and Community Practice Collaborative Care Model for Metastatic Breast Cancer Care (Project ADAPT): Protocol for an Implementation Science–Based Study
Source: JMIR Res Protoc. 2022 Jul 25;11(7):e35736. doi: 10.2196/35736 (PMC9361152; doi:10.2196/35736)
Supplement: Multimedia Appendix 3 [file resprot_v11i7e35736_app3.doc]

*Page 1*

Thank you for returning to the ADAPT study.

In this survey, some of the questions below were asked the last time we contacted you. We want to know if there has been any change to some of the information you provided the last time so we can get a better understanding of the people completing this survey.

Date

__________________________________

(mm-dd-yyyy)

1. Has there been a change to your health insurance
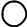
 Yes

status?
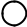
 No

What is your current health Insurance?


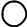
 Medicaid


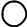
 Medicare


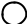
 Employer-based insurance


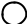
 Tri-Care


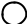
 Union-based


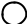
 Self pay (purchase insurance on own)


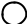
 Uninsured (no insurance)


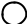
 Prefer to self-describe


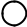
 Prefer not to answer

Prefer to self-describe my health insurance as

|  | __________________________________ |
| --- | --- |
|  |  |
| 2. Has there been a change to your relationship | Yes |
| status? | No |
|  |  |
| What is your current relationship status? | Single |
|  | Married |
|  | Divorced |
|  | Widowed |
|  | Living with significant other. |
|  | Separated |
|  | Prefer not to Answer. |
|  |  |
| 3. Do you have a primary care physician? | Yes |
|  | No |
|  | Prefer not to answer |
|  |  |
| 4. Primary language spoken at home | English |
|  | Spanish |
|  | Prefer to self-describe |
|  | Prefer not to answer |
|  |  |
| Please specify the primary language you speak at home | __________________________________ |
|  |
|  |  |
| 5. Has there been a change to your level of formal | Yes |
| education completed? | No |


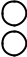

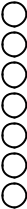

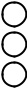

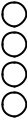

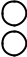


*Page 2*

| What level of formal education have you currently | 8th grade or less |
| --- | --- |
| completed? | Some high school (HS) |
|  | Graduated HS or GED |
|  | Vocational school |
|  | Associate degree |
|  | Bachelor's degree |
|  | Graduate or professional degree |
|  | Prefer not to answer |
|  |  |
| 6. Has there been a change to your employment status? | Yes |
|  | No |
|  |  |
| What is your current employment status? | Full time |
|  | Part time |
|  | Retired |
|  | Unemployed |
|  | Prefer not to answer |
|  |  |
| 7. Has there been a change to the number of jobs you | Yes |
| have? | No |
|  |  |
| How many jobs do you have currently? | __________________________________ |
|  |
|  |  |
| 8. Has there been a change to your yearly family | Yes |
| income, including all sources? | No |
|  |  |
| What is your yearly family income, including all | Less than $15,000 |
| sources? | $15,00 -$34,999 |
|  | $35,000-54,999 |
|  | $55,000-$74,999 |
|  | $75,000 or more |
|  | Prefer not to answer |
|  |  |
| 9. Would you say that in general your health is | Excellent |
|  | Very good |
|  | Good |
|  | Fair |
|  | Poor |
|  | Prefer not to answer |
|  |  |
| 10. How often do you have someone help you read | All the time |
| hospital materials? | Most of the time |
|  | Some of the time |
|  | A little of the time |
|  | None of the time |
|  | Prefer not to answer |
|  |  |
| 11. How confident are you filling out medical forms by | Extremely |
| yourself? | Quite a bit |
|  | Somewhat |
|  | A little bit |
|  | Not at all |
|  | Prefer not to answer |


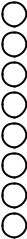

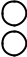

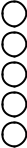

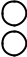

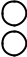

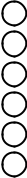

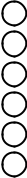

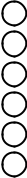

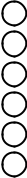


*Page 3*

| 12. How often do you have problems learning about your | All the time |
| --- | --- |
| medical condition because of difficulty understanding | Most of the time |
| written information? | Some of the time |
|  | A little of the time |
|  | None of the time |
|  | Prefer not to answer |
|  |  |
| 13. Has your email address changed? | Yes |
|  | No |
|  |  |
| What is your current email address? | __________________________________ |
|  |
|  |  |
| You did not select an option or provide an answer to a | Yes |
| question(s) above, do you wish to continue? | No |


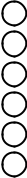

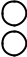

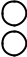


References

Questions 10-12 are the Brief Health Literacy Screener

Chew LD, Bradley KA, Boyko EJ. Brief questions to identify patients with inadequate health literacy. Fam Med. 2004 Sep;36(8):588-94. PMID: 15343421.
