# Supplementary material for: Adapting and Developing an Academic and Community Practice Collaborative Care Model for Metastatic Breast Cancer Care (Project ADAPT): Protocol for an Implementation Science–Based Study
Source: JMIR Res Protoc. 2022 Jul 25;11(7):e35736. doi: 10.2196/35736 (PMC9361152; doi:10.2196/35736)
Supplement: Multimedia Appendix 4 [file resprot_v11i7e35736_app4.doc]

*Page 1*

Please complete the survey below. To be completed by the Partner site research team/ADAPT Coordinator and not the Patient.

Thank you!

Date

|  | __________________________________ | |
| --- | --- | --- |
|  | (mm-dd-yyyy) | |
|  |  |  |
| Patient's First name | __________________________________ | |
|  |
|  |  |  |
| Patient's Last name | __________________________________ | |
|  |
|  |  | |
| Patient study phase | Exploration | |
|  | T0 | (Enrollment) |
|  | T2 | (6 month) |


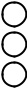


**Receptor status**

**Estrogen receptor**

Estrogen receptor evaluation
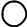
 Not done


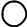
 Done


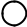
 Unknown

Estrogen receptor result
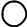
 Negative


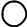
 Positive


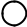
 Undetermined

Estrogen receptor actual value (%)

__________________________________

Estrogen receptor Allred Score

__________________________________

**Progesterone receptor**

Progesterone receptor evaluation
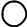
 Not done


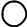
 Done


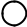
 Unknown

Progesterone receptor result
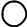
 Negative


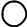
 Positive


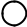
 Undetermined

Progesterone receptor actual value (%)

__________________________________

Progesterone receptor Allred Score

__________________________________

*Page 2*

**Her2**

HER2 evaluation

Not done


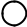


Done


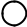


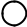
 Unknown

HER2 result

Negative


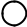


Positive


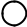


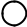
 Indeterminate

Type of HER2 test

IHC


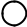


FISH


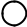


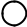
 Unknown

**Diagnosis date and type**

Date of diagnosis of metastatic disease

__________________________________

De Novo?

Yes


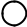


No


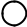


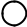
 Unknown

Recurrent metastatic disease?

Yes


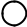


No


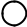


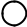
 Unknown

**Treatment**

Number of PRIOR lines of therapy (enter number)

__________________________________

First line of therapy agent (enter name)

__________________________________

Second line of therapy agent (enter name if received)

__________________________________

Third line of therapy agent (enter name if received)

__________________________________

CURRENT line of therapy agent (enter line and name)

__________________________________
