# Supplementary material for: Adapting and Developing an Academic and Community Practice Collaborative Care Model for Metastatic Breast Cancer Care (Project ADAPT): Protocol for an Implementation Science–Based Study
Source: JMIR Res Protoc. 2022 Jul 25;11(7):e35736. doi: 10.2196/35736 (PMC9361152; doi:10.2196/35736)
Supplement: Multimedia Appendix 5 [file resprot_v11i7e35736_app5.doc]

**Decision Regret Scale**

**Measure can be found at:**

Brehaut JC, O'Connor AM, Wood TJ, Hack TF, Siminoff L, Gordon E, et al. Validation of a decision regret scale. Med Decis Making 2003;23(4):281-292. doi: 10.1177/0272989X03256005. Medline: 12926578.

The Ottawa Hospital Research Institute: https://decisionaid.ohri.ca/eval_regret.html
