# Supplementary material for: Adapting and Developing an Academic and Community Practice Collaborative Care Model for Metastatic Breast Cancer Care (Project ADAPT): Protocol for an Implementation Science–Based Study
Source: JMIR Res Protoc. 2022 Jul 25;11(7):e35736. doi: 10.2196/35736 (PMC9361152; doi:10.2196/35736)

*Page 1*

This survey focuses on your satisfaction as a medical provider with the referral process of patients needing cancer care.

Please complete the ADAPT Provider survey below.

Thank you.

Date of survey

__________________________________

(mm-dd-yyyy)

**Thank you for agreeing to complete this survey today. Below, we're going to ask some questions about how you care for patients with metastatic breast cancer. We would like to learn more about your experience with receiving patients with metastatic breast cancer referred from a community hospital to Siteman Cancer Center (SCC). We would also like to learn about your preferred means of communication regarding your patients' cancer care.**

**On a scale from "Not at all satisfied" to "Extremely satisfied", please rate the following below**

| Not at all | Slightly satisfied | Moderately | Very satisfied | Extremely |
| --- | --- | --- | --- | --- |
| satisfied |  | satisfied |  | satisfied |

1. What is your overall


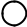

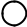

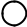

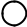

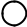


satisfaction with the initial

metastatic breast cancer

consultation of outside referrals

at your site?

Why weren't you satisfied with the initial consult?

__________________________________________

**On a scale from "Not a barrier" to "Significant barrier", please rate the following clinical barriers you have noticed when receiving patients referred from a community hospital to Siteman Cancer Center (SCC).**

Not a barrier Somewhat of a barrier Moderate barrier Significant barrier

2. Administrative support


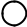

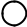

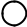

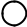


3. Patient tracking system


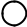

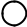

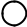

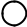


4. Availability of medical records


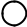

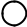

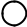

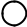


5. Time constraints in your clinic


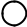

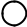

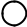

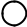


6. Referring provider interest in


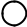

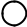

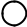

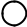


referring a patient to SCC

7. Contacting referring provider


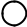

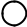

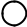

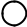


*Page 2*

8. Referring provider contacting


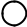

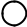

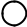

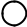


you

9. Patient interest in a referral to


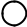

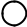

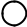

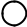


SCC

10. Patient willingness to travel


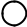

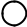

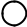

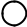


to SCC

11. Long wait time for date of


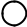

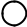

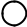

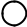


initial appointment at SCC

12. Lack of knowledge of


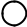

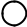

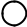

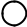


available clinical trials at SCC

For any barrier question, why were these such

barriers?

__________________________________________

**On a scale from "Not at all important" to "Extremely important", how important are the following in referring a patient?**

| Not at all | Slightly | Moderately | Very important | Extremely |
| --- | --- | --- | --- | --- |
| important | important | important |  | important |

13. Access to referring provider


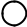

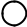

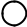

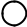

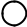


email

14. Access to referring provider


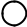

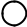

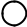

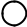

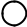


phone numbers

15. Communication between


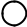

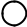

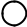

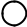

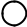


administrative/research staff

regarding the patient referral

and follow-up visits

16. EPIC messaging between


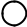

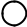

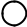

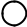

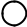


providers

17. Communication between


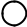

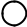

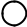

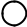

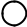


clinical staff to clinical staff

(nurse coordinator)

18. Availability of a SCC


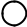

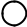

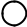

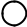

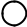


telemedicine appointment for

the patient

**On a scale from "Not at all satisfied" to "Extremely satisfied", how satisfied are you with the following below with regards to referring MDs?**

| Not at all | Slightly satisfied | Moderately | Very satisfied | Extremely |
| --- | --- | --- | --- | --- |
| satisfied |  | satisfied |  | satisfied |

19. Access to referring provider


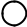

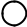

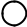

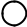

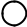


email

20. Access to referring provider


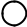

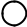

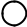

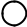

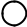


phone number

*Page 3*

21. Communication between


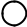

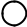

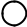

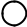

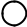


administrative/research staff

regarding the patient referral

and follow-up visits

| 22. What is your preferred method of communication? Do | MD to MD |
| --- | --- |
| you want this to be: (you can select more than one | nurse to nurse |
| option) | Other staff (admin) to Other staff (admin) |
|  |  |
| 23. What other experiences or barriers with the |  |
| referral process would you like to share? | __________________________________________ |
|  |
|  |  |
| You did not select an option or provide an answer to a | Yes |
| question(s) above. Do you wish to continue? | No |


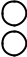

Supplement: Multimedia Appendix 6 [file resprot_v11i7e35736_app6.doc]
