# Supplementary material for: Adapting and Developing an Academic and Community Practice Collaborative Care Model for Metastatic Breast Cancer Care (Project ADAPT): Protocol for an Implementation Science–Based Study
Source: JMIR Res Protoc. 2022 Jul 25;11(7):e35736. doi: 10.2196/35736 (PMC9361152; doi:10.2196/35736)
Supplement: Multimedia Appendix 7 [file resprot_v11i7e35736_app7.doc]

*Page 1*

This survey focuses on your satisfaction as a medical provider with the referral process of patients needing cancer care.

Please complete the ADAPT Provider survey below.

Thank you.

Date of survey

__________________________________

(mm-dd-yyyy)

**Thank you for agreeing to complete this survey today. Below are some questions about how you care for patients with metastatic breast cancer (MBC). We would like to learn more about your experience referring patients with metastatic breast cancer from your hospital to Siteman Cancer Center (SCC). We would also like to learn about your preferred means of communication regarding your patients' cancer care.**

**On a scale from "Not at all satisfied" to "Extremely satisfied", please rate the following below**

| Not at all | Slightly | Moderately | Very satisfied | Extremely | Not applicable |
| --- | --- | --- | --- | --- | --- |
| satisfied | satisfied | satisfied |  | satisfied |  |

1. What is your overall


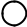

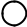

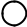

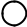

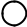

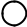


satisfaction with the

communication with SCC MD

prior to an in person consult

(virtual consult with providers,

telemedicine consults for

patient)?

2. What is your overall


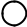

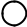

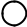

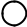

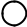

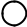


satisfaction with the SCC

in-person consult?

Why weren't you satisfied with the communication prior

to the in person consult?

__________________________________________

Why weren't you satisfied with the in person consult

at Siteman?

__________________________________________

**On a scale from "Not a barrier" to "Significant barrier", please rate the following clinical barriers in referring patients from your institution (Note: SCC is Siteman Cancer Center).**

Not a barrier Somewhat of a barrier Moderate barrier Significant barrier

3. Administrative support


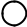

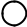

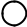

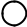


*Page 2*

4. Patient tracking system


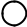

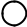

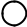

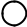


5. Availability of medical records


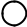

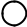

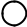

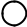


6. Time constraints in your clinic


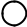

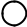

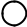

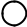


7. Your interest in a referring a


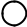

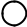

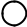

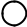


patient to SCC

8. Your experience contacting


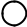

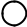

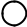

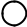


SCC to refer a patient

9. Patient interest in a referral to


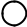

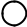

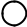

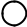


SCC

10. Patient willingness to travel


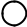

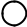

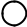

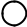


to SCC

11. Long wait time for date of


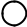

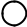

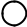

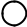


initial appointment at SCC

12. Lack of knowledge of


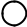

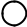

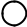

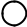


available clinical trials at SCC

For any barrier question, why were these such

barriers?

__________________________________________

**On a scale from "Not at all important" to "Extremely important", how important are the following in referring a patient?**

| Not at all | Slightly | Moderately | Very important | Extremely |
| --- | --- | --- | --- | --- |
| important | important | important |  | important |

13. Access to SCC providers'


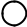

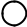

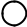

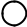

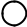


email

14. Access to SCC providers'


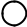

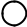

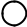

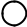

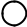


phone number

15. Communication between


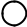

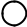

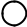

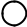

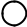


administrative/research staff

regarding the patient referral

and follow-up visits

16. EPIC messaging between


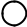

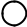

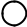

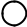

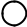


providers

17. Communication between


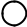

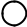

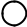

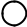

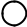


clinical staff to clinical staff

(nurse coordinator)

18. Availability of a SCC


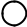

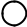

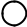

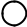

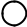


telemedicine appointment for

the patient

*Page 3*

**On a scale from "Not at all satisfied" to "Extremely satisfied", how satisfied are you with the following below?**

| Not at all | Slightly satisfied | Moderately | Very satisfied | Extremely |
| --- | --- | --- | --- | --- |
| satisfied |  | satisfied |  | satisfied |

19. Access to SCC providers'


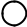

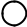

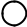

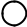

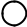


email

20. Access to SCC providers'


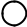

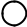

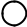

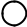

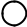


phone number

21. Communication between


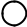

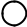

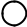

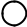

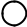


administrative/research staff

regarding the patient referral

and follow-up visits

| 22. What is your preferred method of communication? Do | MD to MD |
| --- | --- |
| you want this to be: (you can select more than one | nurse to nurse |
| option) | Other staff (admin) to Other staff (admin) |

**On a scale from "Not at all satisfied" to "Extremely satisfied”, kindly rate the following below (Note: SCC is Siteman Cancer Center).**

| Not at all | Slightly satisfied | Moderately | Very satisfied | Extremely |
| --- | --- | --- | --- | --- |
| satisfied |  | satisfied |  | satisfied |

23. Knowledge of supportive


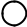

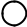

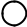


care and educational resources

at SCC

24. knowledge of clinical trials at

SCC

25. Current discussion and

coordination of testing with SCC

MDs prior to patient referral

For any of the knowledge questions, why were you not

satisfied?

|  | __________________________________________ |
| --- | --- |
|  |  |
| 26. At what point during treatment do you typically | First line |
| refer patients to clinical trials? | Second line |
|  | Third line |
|  | Beyond |
|  | Prefer not to answer |
|  |  |
| 27. Do you typically refer prior to starting next line | Before |
| therapy or after? | After |
|  | About evenly between the two |
|  | Prefer not to answer |

*Page 4*

**As a referring provider, we would like to learn more about the various means in which you communicate or preferred means of communication with SCC providers regarding your patients' cancer care.**

**How do you communicate interest in clinical trials for your patient?**

No Yes Not applicable

28. Call the new patient intake

office at SCC

29. Email the new patient intake

office at SCC

30. Email research nurse at SCC

31. Email a SCC MD

32. Call a SCC MD

33. Email a study clinical

research coordinator at SCC

34. SCC website

1. Please specify other way(s) you communicate interest in clinical trials for your patients:

__________________________________________

1. What other experiences or barriers with the referral process would you like to share?

__________________________________________

You did not select an option or provide an answer to a question(s) above, do you wish to continue?

Yes

No
