# Supplementary material for: Adapting and Developing an Academic and Community Practice Collaborative Care Model for Metastatic Breast Cancer Care (Project ADAPT): Protocol for an Implementation Science–Based Study
Source: JMIR Res Protoc. 2022 Jul 25;11(7):e35736. doi: 10.2196/35736 (PMC9361152; doi:10.2196/35736)
Supplement: Multimedia Appendix 8 [file resprot_v11i7e35736_app8.doc]

*Page 1*

**Implementation Climate Scale (ICS)**

Measure can be found at:

Ehrhart MG, Aarons GA, Farahnak LR. Assessing the organizational context for EBP implementation: the development

and validity testing of the Implementation Climate Scale (ICS). Implement Sci 2014 Oct 23;9:157. doi: 10.1186/s13012-014-0157-1. Medline: 25338781.
