# Supplementary figures and images for: Adapting and Developing an Academic and Community Practice Collaborative Care Model for Metastatic Breast Cancer Care (Project ADAPT): Protocol for an Implementation Science–Based Study
Source: JMIR Res Protoc. 2022 Jul 25;11(7):e35736. doi: 10.2196/35736 (PMC9361152; doi:10.2196/35736)

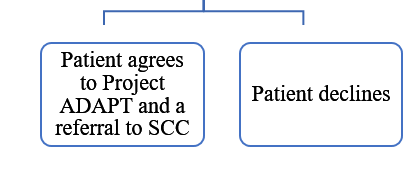

Supplement: Multimedia Appendix 10 [file resprot_v11i7e35736_app10.docx]
