# Supplementary material for: Adapting and Developing an Academic and Community Practice Collaborative Care Model for Metastatic Breast Cancer Care (Project ADAPT): Protocol for an Implementation Science–Based Study
Source: JMIR Res Protoc. 2022 Jul 25;11(7):e35736. doi: 10.2196/35736 (PMC9361152; doi:10.2196/35736)
Supplement: Supplementary file 10 [file resprot_v11i7e35736_app9.docx]

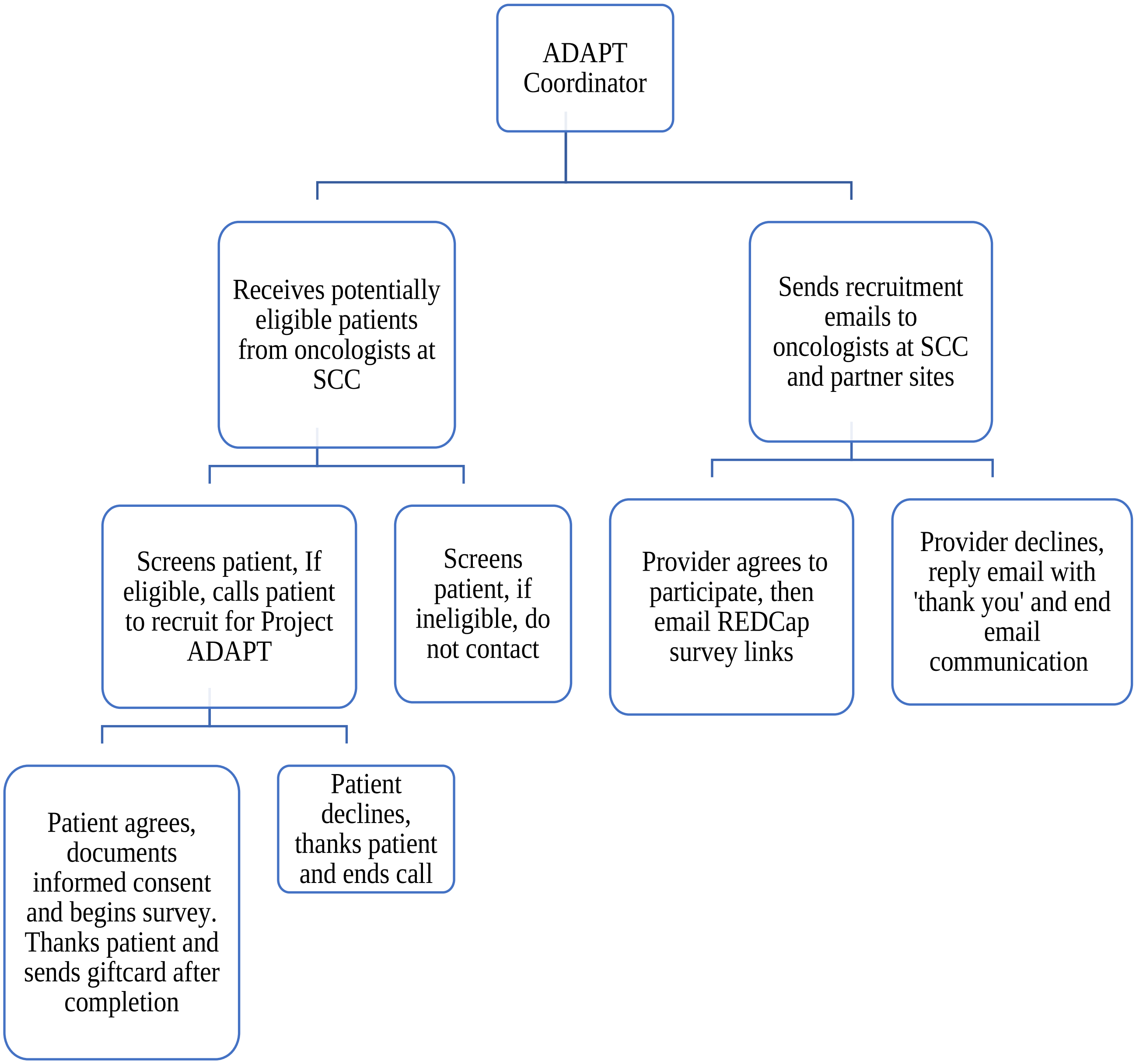
ADAPT Coordinator
Receives potentially eligible patients from oncologists at SCC
Sends recruitment emails to oncologists at SCC and partner sites
Screens patient, If eligible, calls patient to recruit for Project ADAPT
Screens patient, if ineligible, do not contact
Provider agrees to participate, then email REDCap survey links
Provider declines, reply email with 'thank you' and end email communication
Patient agrees, documents informed consent and begins survey. Thanks patient and sends giftcard after completion
Patient declines, thanks patient and ends call
